# Newborn Screening for X-Linked Adrenoleukodystrophy: The Initial Illinois Experience

**DOI:** 10.3390/ijns8010006

**Published:** 2022-01-17

**Authors:** Barbara K. Burton, Rachel Hickey, Lauren Hitchins, Vera Shively, Joan Ehrhardt, Laura Ashbaugh, Yin Peng, Khaja Basheeruddin

**Affiliations:** 1 Department of Pediatrics, Ann and Robert H. Lurie Children’s Hospital of Chicago, Chicago, IL 60611, USA; rahickey@luriechildrens.org (R.H.); lhitchins@luriechildrens.org (L.H.); VShively@luriechildrens.org (V.S.); 2 Department of Pediatrics, Feinberg School of Medicine, Northwestern University, Chicago, IL 60611, USA; 3 Office of Health Promotion, Illinois Department of Public Health, Springfield, IL 62761, USA; Joan.Ehrhardt@Illinois.gov (J.E.); Laura.Ashbaugh@Illinois.gov (L.A.); 4 Newborn Screening Laboratory, Illinois Department of Public Health, Chicago, IL 60612, USA; Yin.Peng@Illinois.gov (Y.P.); Khaja.Basheeruddin@Illinois.gov (K.B.)

**Keywords:** X-linked adrenoleukodystrophy, peroxisomal disorder, newborn screening

## Abstract

X-linked adrenoleukodystrophy (X-ALD) is a genetic neurodegenerative disorder with an approximate incidence of 1 in 14,700 births. Both males and females are affected. Approximately one-third of affected males develop childhood cerebral adrenoleukodystrophy, which progresses rapidly to severe disability and death. In these cases, early surveillance and treatment can be lifesaving, but only if initiated before the onset of neurologic symptoms. Therefore, X-ALD was added to the Recommended Uniform Screening Panel. We report outcomes of the initial screening of approximately 276,000 newborns in Illinois. The lipid C26:0 lysophosphatidylcholine (C26:0-LPC) was measured in dried blood spots (DBS) using liquid chromatography with tandem mass spectrometry. Results ≥ 0.28 µmol/L were considered screen positive. Of 18 screen positive results detected, 12 cases were confirmed. Results were reported as borderline if initial and repeat analyses were ≥0.18 and <0.28 µmol/L. Of the 73 borderline screen results, 57 were normal after analysis of a second sample. Five X-ALD cases were identified from borderline screens. Newborn screening of X-ALD was successfully implemented in Illinois, and results were comparable to reports from other states. Early identification of infants with this potentially life-threatening disorder will significantly improve outcomes for these children.

## 1. Introduction

X-linked adrenoleukodystrophy (X-ALD) is a genetic neurodegenerative disorder resulting from mutations in the *ABCD1* gene. It has an estimated incidence in the United States population of 1 in 14,700 births [1]. The disorder is associated with impaired peroxisomal oxidation of very-long-chain fatty acids (VLCFA) and VLCFA elevations as measured in plasma. Both males and females are affected, and a range of phenotypes are observed in affected individuals, with no genotype–phenotype correlation [2]. The most common phenotype is adrenomyeloneuropathy, which is characterized by progressive weakness, spasticity, and bowel and bladder dysfunction in adult life. However, approximately one-third of affected males develop rapidly progressive brain involvement, referred to as childhood cerebral adrenoleukodystrophy (CCALD) at 3–10 years of age with a smaller fraction developing a cerebral disease in adolescence or adult life. In contrast, the cerebral disease is rare in females. When cerebral disease occurs, it progresses rapidly to severe disability and death. Addison’s disease is common in affected males as well and can be the earliest or only sign of the disorder [3].

In patients known to have X-ALD, surveillance for Addison’s disease and early treatment can be lifesaving. In addition, hematopoietic stem cell transplantation (HSCT) has been shown to be effective in arresting the progression of cerebral adrenoleukodystrophy, but only if performed prior to the onset of neurologic symptoms and before advanced brain involvement is seen on neuroimaging [4]. Follow-up of known affected individuals with serial magnetic resonance imaging (MRI) of the brain can allow recognition of the early signs of cerebral disease at a time when transplantation can be successfully performed and the outcome improved. For this reason, X-ALD was added to the Recommended Uniform Screening Panel (RUSP) by the Advisory Committee on Heritable Disorders in Newborns and Children and accepted by the Secretary of Health and Human Resources in 2016 [5]. Since that time, newborn screening has been implemented in a number of states with plans for implementation in many others.

The purpose of this report is to describe the outcome of the initial experience with X-ALD newborn screening in the state of Illinois.

## 2. Materials and Methods

Statewide newborn screening for X-ALD began on 18 June 2019 in the Illinois Department of Public Health. Samples from all Illinois birth hospitals were transported to the Newborn Screening Laboratory in Chicago for analysis. Testing was performed by measuring C26:0 lysophosphatidylcholine (C26:0-LPC) in 3.2 mm dried blood spots (DBS) using negative ion-mode liquid chromatography–tandem mass spectrometry on a QSight^®^210MD mass spectrometer. After adding 0.36 μM of a stable-isotope-labeled internal standard, C26:0-d4LPC in 100 μL methanol, 96-well plates were incubated at room temperature for 30 min. The extracted lipids were transferred into assay plates after centrifugation at 3000 rpm for 5 min. The internal standard was purchased from PerkinElmer^®^. Each sample was injected into the mass spectrometer by isocratic separation on an HPLC column (Xterra^®^MS C8 Vanguard^®^cartridge, 125A, 3.5 μm, 2.1 mm × 5 mm) and individually evaluated by measuring the concentration of the internal standard, C26:0-LPC and C24:0-LPC [6]. The lipid concentration is expressed as µmol/L.

An initial screen for ALD was performed in a single punch and, if the C26:0-LPC levels were ≥ 0.16 µmol/L, the analysis was repeated in triplicate. The results above the positive cutoff (≥ 0.28 µmol/L) were reported as positive. Immediate referral for diagnostic testing was recommended. Samples from newborns with results ≥ 0.18 and < 0.28 µmol/L were reported as borderline, and a second DBS specimen would be requested in this case. If the initial and repeat results on the same DBS specimen were not consistent, or the C24:0-LPC concentration was below 0.02 µmol/L, the specimen result was deemed invalid, with a request for specimen resubmission. Samples < 0.18 μmol/L were reported as screen negative.

The Newborn Screening Laboratory notified the follow-up program staff of any positive, borderline, or invalid test results at which time the ordering physician was contacted with a recommendation for either a repeat sample or referral, as indicated. Physicians were provided with a list of designated consultants who can provide diagnostic testing and treatment of affected infants. Follow-up testing on infants with a positive newborn screen was at the discretion of the designated consultants and not dictated by protocol. Typically, this began with the measurement of plasma VLFCAs. If this was normal, no further testing may be conducted, and the case was classified as a false positive or as normal. In the event of elevation of VLCFA, *ABCD1* gene sequencing was performed on both male and female infants. If this was normal, sequencing of peroxisomal genes may be conducted. A diagnosis of X-ALD was generally considered confirmed if the VLCFAs were consistent with this diagnosis and a known pathogenic variant, likely pathogenic variant or VUS was detected in the *ABCD1* gene. Additional confirmation was obtained in most cases by demonstration of elevated plasma VLCFAs in the mother as well. Data on the results of follow-up testing for screen positive infants were forwarded from consultants to the follow-up program staff.

The data from the time period covered by this report were provided in a de-identified format to one of the authors (Barbara K. Burton) per Institutional Review Board (IRB) guidelines and a data transfer agreement between the Illinois Department of Public Health and the author’s institution. Prior to any transmittal of data, the project was approved by the IRB of the Illinois Department of Public Health. The Illinois Department of Public Health routinely sends out short-term follow-up forms to designated consultants to gather data on follow-up testing and final diagnosis. For affected infants, long-term follow-up forms are sent out on an annual basis.

In the case of diagnosed infants, evaluation of the infant and other family members was at the discretion of the designated consultant and not directed by protocol. Once a diagnosis was established in an infant, follow-up was also at the discretion of the treating physician, but for male infants, this was generally accomplished according to published recommendations for both MRI surveillance and screening for adrenal insufficiency. No testing or screening was typically performed on female infants, but genetic counseling was provided. Cascade testing of siblings and mothers was routinely performed and offered for other family members at risk following pedigree analysis [7,8].

## 3. Results

A total of 306,929 specimens were analyzed between 18 June 2019 and 31 May 2021, representing approximately 276,000 individual newborns. There were 18 individuals (7 males; 11 females) who had an initial positive screening test result and were referred for diagnostic evaluation. Of the males, four were found to be affected with X-ALD, one was diagnosed with a peroxisomal biogenesis disorder (*PEX1* mutations), one was normal on follow-up testing, and data are pending for one. Of the eleven females, seven were found to be heterozygotes for X-ALD, two were diagnosed with other peroxisomal disorders (one D-bifunctional protein deficiency and one unspecified), one was homozygous for an *ABCD1* mutation due to isodisomy X and classified as affected with X-ALD, and one is pending. Of the 16 positive cases for which follow-up data were received, one was a false positive. In this case, VLCFAs were normal on follow-up.

**Figure 1:**
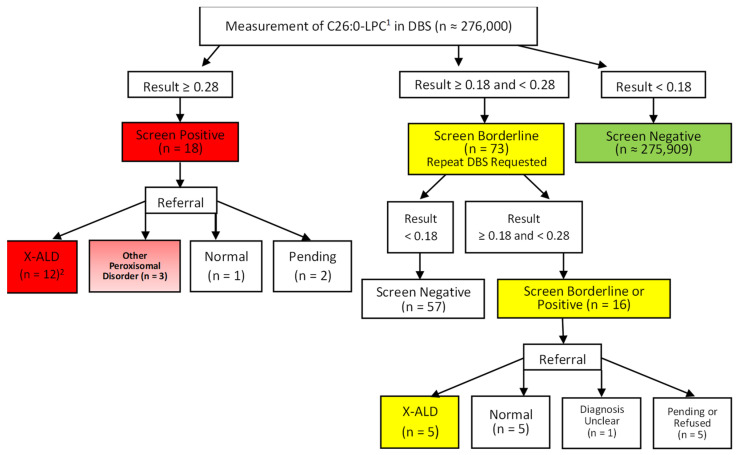
Outcome of newborn screening for X-ALD in Illinois from 18 June 2019 to 31 May 2021. ^1^ Values for C26:0-LPC are expressed as μmol/L. ^2^ Value for n includes four males, seven female heterozygotes, and one female homozygous for *ABCD1* mutation due to isodisomy X.

In addition to the 18 infants with an initial positive result, 73 infants had an initial borderline result. Repeat DBS specimens were requested, and the results were normal in 57 cases. The remaining 16 had results that were again in the borderline range or positive on the subsequent specimen and were referred for diagnostic testing. Of these 16 infants, 3 males were diagnosed with X-ALD, and 2 female infants were found to be heterozygotes for X-ALD. Five infants had normal results, including four based on follow-up testing and one premature infant who died before any diagnostic testing was performed but had a third DBS specimen submitted with a normal result. One female had mildly elevated VLCFA with no detectable mutation in *ABCD1* and the diagnosis remains unclear at the last follow-up. Finally, one family refused further testing, and four cases are pending. A summary of the results of newborn screening for X-ALD in all infants in this series is diagrammed in Figure 1.

A total of seven males, one female homozygous due to isodisomy X, and nine female heterozygotes with X-ALD were identified for an approximate combined incidence of 1 in 16,200 births in this cohort. Molecular variants detected in these cases are specified in Table 1. Three other peroxisomal disorders were identified (1 in 92,000 births). Overall, 91 of 276,000 infants (0.03%) had a positive or borderline result requiring some type of further testing.

The mean C26:0-LPC result on the initial DBS sample for males with X-ALD was 0.30 with a range of 0.21–0.43 and for female heterozygotes, it was 0.30 (range 0.18–0.44).

The three infants with other peroxisomal disorders had initial C26:0-LPC levels of 0.78, 0.94, and 1.72, all significantly higher than observed in any male or female heterozygote with X-ALD. The female who was homozygous for an *ABCD1* mutation due to isodisomy X had a C26:0-LPC level of 0.76, similar to the values seen in the patients with other peroxisomal disorders. Considering only diagnoses of X-ALD and assuming all pending or unresolved cases are normal, the positive predictive value of an initial positive screen was 67% (12 of 18), while the positive predictive value of a borderline screen was 6.8% (5 of 73).

Only two specimens were reported as invalid. The repeat DBS specimens were normal.

## 4. Discussion

The experience with X-ALD newborn screening described here from Illinois parallels that reported earlier from other states [9,10]. Although the initial experience in Minnesota suggested that the incidence of X-ALD might be greater than previously estimated, our incidence of approximately 1 in 16,200 births is comparable to previous estimates and very close to that observed in New York State [9]. Screening for this disorder has broad implications that go beyond the affected infant since older siblings, mothers, and other family members are often found to be affected through family cascade testing. Many of these other individuals will also benefit from surveillance and early treatment if Addison’s disease or cerebral involvement is detected. In addition to identifying infants with X-ALD, measurement of C26:0-LPC also leads to the diagnosis of infants with other peroxisomal disorders, which, while much less common, are also associated with impaired VLCFA metabolism but exhibit a very different and variable phenotype. One in 92,000 infants in our series was found to have a different peroxisomal disorder.

The false-positive rate associated with newborn screening for X-ALD in this series was low. A total of 91 infants out of 276,000 screened had an initial positive or borderline result requiring some additional testing. In 57 cases, a repeat DBS determination was normal, and no further evaluation or testing was required. Overall, 33 infants were referred to consultants for diagnostic testing, which excludes the infant who expired before referral. Of these, 17 were diagnosed with X-ALD and 3 with a different peroxisomal disorder. Even if it is assumed that all the pending or unresolved cases are normal, this means that 52% of referred infants had a positive diagnosis.

We found that the patients with other peroxisomal disorders had C26:0-LPC levels on newborn screening that were significantly higher than those observed in male or female infants with X-ALD. The exception was one female who was homozygous for the *ABCD1* mutation due to isodisomy X. While cerebral involvement and adrenal insufficiency are generally rare in females with X-ALD, cerebral involvement has been reported in conjunction with skewed X-inactivation [11]. Therefore, this female is presumed to be at elevated risk and is being followed according to recommendations for affected males.

## 5. Conclusions

In summary, newborn screening for X-ALD was successfully implemented in the state of Illinois. The follow-up of screen-positive infants yielded results comparable to those reported from other states. It is anticipated that early recognition and surveillance of infants with this potentially life-threatening disorder will have a positive impact on the child and family and will significantly improve the outcome.

## Figures and Tables

**Table 1:** Molecular variants detected in confirmed X-ALD cases.

Case Number	Sex	Screening Test Result	*ABCD1* Sequence Variant	Variant Classification by Laboratory
1	Male	Borderline	c.582C>G, p.D194E	VUS ^1^
2	Female	Borderline	c.839G>A, p.R280H	VUS
3	Male	Borderline	c.1900G>A, p.A634T	VUS
4	Female	Borderline	c.877G>A, p.E292K	VUS
5	Male	Borderline	c.853C>A, p.R285S	VUS
6	Female ^2^	Positive	c.1166 G>A, p.R389H	Pathogenic
7	Male	Positive	None ^3^	Pending ^3^
8	Female	Positive	c.751_753 del	Pathogenic
9	Female	Positive	c.626C>T	VUS
10	Female	Positive	c.9del (p.Leu4Serfs*I2)	Pathogenic
11	Female	Positive	c.1876G>A, p.A626T	Pathogenic
12	Female	Positive	Not tested ^4^	Not known
13	Male	Positive	c.838C>T, p.R280C	Pathogenic
14	Female	Positive	c.1747G>A	VUS
15	Female	Positive	c.1166G>A, p.R389H	Pathogenic
16	Male	Positive	c.839G>A, p.R280H	VUS
17	Male	Positive	c.839G>A, p.R280H	Pathogenic

^1^ Variant of uncertain significance. ^2^ Female Case Number 6 is homozygous for the *ABCD1* variant specified due to isodisomy X. All other females were heterozygous for the variant shown. ^3^ No *ABCD1* mutation; deletion/duplication and whole-exome sequencing pending. ^4^ Not tested; mother, known X-ALD carrier.

## Data Availability

The data presented in this study are available on request from the corresponding author. The data are not publicly available due to privacy concerns.
